# Influence of Pd Coating Thickness and Pd Content in Sn-Based Solders on Interfacial IMC Formation and Microstructural Evolution in Solder/Ni Joints

**DOI:** 10.3390/ma19030526

**Published:** 2026-01-28

**Authors:** Chao-Hong Wang, Chu-An Li, Kuan-Ting Li, Hsuan-Wei Chiu

**Affiliations:** Department of Chemical Engineering, National Chung Cheng University, Chiayi 621301, Taiwan

**Keywords:** interfacial reactions, ENEPIG, diffusion barrier, Pd coating thickness

## Abstract

Interfacial reactions between Sn-based solders and Au/Pd/Ni metallization were investigated at 260 °C, with particular emphasis on the effects of Pd and Sn thicknesses. Au/Pd/Ni substrates with Pd layers of approximately 70 nm, 200 nm, and 1 µm were reacted with Sn layers of about 50, 20, and 10 µm. Additionally, Sn-Pd and Sn-3Ag-Pd solders containing 0.1–1 wt.% Pd were reacted with Ni substrates. In the Sn/Au/Pd/Ni reactions, rapid dissolution of the Pd layer and partial Ni dissolution at the early stage promoted the formation of large amounts of faceted (Pd,Ni)Sn_4_. With increasing reaction time, continuous Ni diffusion enriched the interfacial region, leading to the nucleation and growth of Ni_3_Sn_4_. Once the Ni solubility limit in (Pd,Ni)Sn_4_ was exceeded, this phase gradually transformed into the thermodynamically more stable Ni_3_Sn_4_. In addition to phase evolution, Pd was found to significantly influence the interfacial grain morphology. Minor Pd additions enhanced the Ni_3_Sn_4_ nucleation, resulting in refined and columnar grains. In the Sn-Pd/Ni reactions, low Pd contents led to the rapid replacement of (Pd,Ni)Sn_4_ by Ni_3_Sn_4_, whereas higher Pd contents significantly enhanced the stability and interfacial retention of (Pd,Ni)Sn_4_. These results reveal that increasing Pd thickness or Pd content in the solder significantly enhances the stability of (Pd,Ni)Sn_4_, whereas reducing Sn thickness markedly accelerates interfacial reactions and phase transformation. The experimental observations can be consistently interpreted using a local interfacial equilibrium hypothesis based on the Sn-Pd-Ni phase diagram.

## 1. Introduction

The continuous miniaturization of electronic devices and the rapid development of advanced packaging technologies have led to a significant reduction in solder joint dimensions, such as those in flip-chip solder joints and micro-bump interconnections. As the characteristic size of solder joints decreases, interfacial reactions between the solder and surface finish become increasingly critical in determining joint reliability [1,2]. In most electronic packaging processes, solder joints are formed through liquid/solid reactions during reflow, during which rapid dissolution and intermetallic compound (IMC) formation occur [3,4]. Therefore, the selection and design of surface finishes play an important role in controlling interfacial microstructures and ensuring long-term reliability.

Among various surface finishes, electroless Ni/electroless Pd/immersion Au (ENEPIG) has been widely adopted in advanced packaging due to its excellent solderability, corrosion resistance, and compatibility with fine-pitch interconnections [5,6,7]. In the ENEPIG structure, the thin Pd layer is introduced primarily to suppress the formation of the so-called black pad defect by preventing direct interaction between the immersion Au layer and the underlying electroless Ni-P layer during the Au deposition process [8]. The outermost Au layer mainly serves as oxidation protection and dissolves rapidly into molten solder during reflow, while the Ni layer acts as the principal diffusion barrier against Cu substrates [9,10]. Nevertheless, during soldering, the Pd layer would rapidly dissolve into molten Sn, subsequently participating in interfacial reactions [11,12]. This behavior introduces additional complexity to the interfacial reaction mechanisms, particularly with respect to Pd dissolution, the formation of IMCs, and the mutual interactions among Pd, Ni, and Sn. As a result, the presence of Pd may significantly influence interfacial phase formation and microstructural evolution.

Numerous studies have investigated the interfacial reactions of lead-free solder joints with ENEPIG surface finishes [13,14,15,16,17,18,19,20]. In these systems, PdSn_4_ is commonly formed at the interface and typically exhibits plate-like or faceted morphologies [13,14]. Moreover, partial substitution of Pd by Ni in the PdSn_4_ phase has been observed, resulting in the formation of a solid-solution phase denoted as (Pd,Ni)Sn_4_. The formation and stability of this phase are governed by the local chemical environment at the interface, particularly the relative availability of Pd and Ni during interfacial reactions. Since the Pd layer thickness directly controls the amount of Pd available for dissolution into the molten solder, it plays a critical role in determining interfacial reaction behavior [14,15]. Excessive formation of (Pd,Ni)Sn_4_ at the interface has been reported to be detrimental to the reliability of solder joints [16,17,21]. Consequently, the stability of the (Pd,Ni)Sn_4_ phase is dictated by the local balance between Pd dissolution from the surface finish and Ni diffusion from the substrate. Nevertheless, systematic investigations into the combined effects of Pd layer thickness and solder thickness on interfacial reactions and microstructural evolution remain limited. In addition to surface-finish-related factors, the Pd concentration in Sn-based solders also significantly influences the interfacial reactions [21,22]. However, the effect of Pd concentration in the solder on interfacial IMC formation, particularly in reactions with Ni-based substrates, has not yet been fully understood.

In this study, the effects of Pd layer thickness, solder thickness, and Pd concentration on interfacial reactions were systematically investigated to clarify the role of Pd in ENEPIG-based solder joints. In practical ENEPIG systems, the thin Pd layer dissolves rapidly into the molten solder during reflow, and the interfacial reaction is then governed primarily by the interaction between the Pd-containing solder and the underlying Ni layer. To precisely control solder thickness and establish a well-defined Sn/Au/Pd/Ni reaction couple, samples with electroplated Sn layers of 10–50 μm and electroless Pd layers of 70 nm–1 μm were prepared to examine thickness-dependent interfacial behavior. This approach avoids variations associated with solder volume, wetting, and flux effects, enabling a more reliable analysis of interfacial reaction kinetics and phase evolution. In parallel, Sn-based solders containing 0.1–1 wt.% Pd were reacted with Ni substrates to isolate the effect of Pd concentration in the solder. The resulting interfacial microstructures and phase evolution were analyzed to provide insights into Pd-related effects in ENEPIG systems, which are critical for the reliability of advanced micro-scale interconnections.

## 2. Materials and Methods

**Figure 1:**
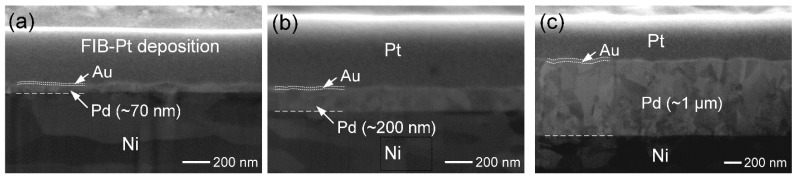
FIB cross-sectional ISE images of Au/Pd/Ni structures with different Pd thicknesses: (**a**) ~70 nm, (**b**) ~200 nm, and (**c**) ~1 µm. In the micrographs, the two dotted lines indicate the Au layer, while the dashed line denotes the Pd/Ni interface.

For the preparation of the Au/Pd/Ni substrates, 0.3 mm thick Ni foils (10 mm × 10 mm, 99.99 wt.%) were first mechanically ground and polished to obtain a flat and smooth surface. The Ni substrates were then subjected to electroless Pd plating using a palladium sulfate (PdSO_4_)-based solution provided by Atotech Taiwan Limited (Taipei, Taiwan). The plating bath was maintained at 55 °C, with a deposition rate of approximately 120 nm per 5 min. Three different Pd-layer thicknesses were prepared by controlling the plating time to 3 min, 12 min, and 50 min, yielding Pd layers of ~70 nm, ~200 nm, and ~1 µm, respectively. Subsequently, a thin Au layer (~20 nm) was deposited on the Pd surface by immersion plating. The thicknesses of the Au and Pd layers were confirmed by cross-sectional milling and observation using a focused ion beam (FIB) combined with ion-induced secondary electron (ISE) imaging, as shown in Figure 1a–c. The Sn layer was then electroplated onto the Au/Pd/Ni substrates at room temperature using an acidic sulfate bath containing SnSO_4_. Three different Sn layer thicknesses, approximately 50 µm, 20 µm, and 10 µm, were prepared by adjusting the electroplating time. The corresponding samples are summarized in Table 1. The interfacial reactions of the Sn/Au/Pd/Ni specimens were carried out at 260 °C for various reaction times.

In addition, the interfacial reactions between Ni substrates and Sn- or Sn-Ag-based solders containing minor Pd additions (0.1, 0.2, 0.5, and 1 wt.%) were investigated. Sn-Pd and Sn-3Ag-Pd solder alloys were prepared from high-purity elemental Sn, Pd, and Ag (99.99 wt.%). The elements were weighed using an electronic balance to obtain a total alloy mass of 2 g and then sealed in evacuated quartz tubes under a vacuum of approximately 10^−2^ Torr. The sealed tubes were annealed at 800 °C for 3 days to ensure complete melting and homogeneity, followed by rapid quenching in water. The solder ingots were cut into discs with a thickness of 1 mm. Ni substrates with dimensions of 5 mm × 5 mm were grounded and polished. A rosin mildly activated (RMA) flux was applied to the Ni substrates, and a piece of the Sn-(Ag)-Pd solder was then placed on each substrate. The assembled reaction couples were heated on a hot plate at 260 °C to conduct the interfacial reactions. After the predetermined reaction times, the samples were rapidly quenched.

The reaction couples were mounted in epoxy resin, followed by grinding and polishing for metallographic examination. The polished samples were lightly etched using a Sn etching solution to enhance the contrast of the interfacial microstructures. Scanning electron microscopy (SEM) operated in back-scattered electron (BSE) imaging mode was employed for microstructural observation. The compositions of the IMC phases were determined by electron probe microanalysis (EPMA, JEOL JXA-8200, JEOL, Tokyo, Japan). In addition, the thickness of the reaction phase was calculated by dividing the measured interfacial area by the corresponding interface length. However, the irregular morphology of the IMC grains makes direct measurement of the layer thickness difficult. Therefore, the average thickness of the IMC layer was estimated by measuring the total interfacial area of the IMC grains over a wide field of view in the BSE images. To ensure accuracy and representativeness, multiple measurements were taken at different locations along the interface, and the reported thickness represents the mean value obtained from these measurements.

## 3. Results and Discussion

### 3.1. Sn/Au/Pd/Ni Reactions

#### 3.1.1. Sn (50 µm)/Au/Pd (70 nm)/Ni System

**Figure 2:**
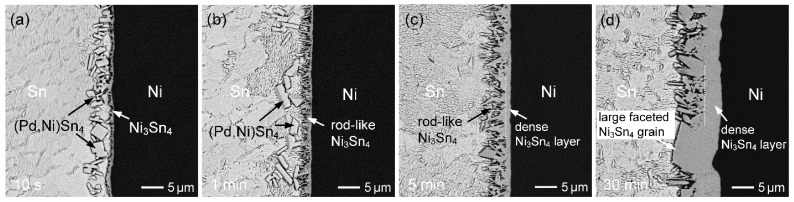
BSE micrographs showing the interfacial microstructures in the Sn (50 µm)/Au/Pd (70 nm)/Ni samples reacted at 260 °C for (**a**) 10 s, (**b**) 1 min, (**c**) 5 min, and (**d**) 30 min.

Figure 2a–d show BSE micrographs illustrating the evolution of interfacial microstructures in the Sn (50 µm)/Au/Pd (70 µm)/Ni system reacted at 260 °C for different reaction times. After a short reaction time of 10 s (Figure 2a), bright plate-like grains together with a thin dark IMC layer were observed at the Sn/Ni interface. EPMA analysis revealed that the bright IMC had a composition of Sn-0.92 at.%Au-4.73 at.%Pd-14.21 at.%Ni. Based on the elemental ratios, this phase can be identified as (Ni,Pd,Au)Sn_4_. Despite the relatively high Ni content in this IMC, NiSn_4_ is considered a metastable phase, rather than an equilibrium phase [23,24]. According to the Sn-Pd-Ni phase diagram reported by Rahman et al. [25], PdSn_4_ exhibits a high Ni solubility of up to ~15.4 at.%. Therefore, this IMC is more appropriately designated as (Pd,Ni)Sn_4_. In addition, the dark IMC layer was identified as Ni_3_Sn_4_ with negligible Pd content. Furthermore, based on the previous study [26], Ni_3_Sn and Ni_3_Sn_2_ can also form, under the solid-state reaction at 125 °C; however, these IMCs are extremely thin (~50 nm) and are therefore not detectable in the SEM observations in this study.

At the early stage of the reaction, a large number of (Pd,Ni)Sn_4_ grains formed in the interfacial zone, accompanied by the formation of Ni_3_Sn_4_ on the Ni side. The interfacial morphology is relatively irregular, suggesting significant dissolution of the Ni substrate. Accordingly, it is inferred that the thin Au/Pd layer was rapidly and completely dissolved into the molten Sn, while partial dissolution of the Ni substrate occurred simultaneously. This resulted in locally elevated Pd and Ni concentrations near the interface, which promoted the precipitation of (Pd,Ni)Sn_4_ grains.

With increasing reaction time to 1 min, as seen in Figure 2b, the (Pd,Ni)Sn_4_ phase gradually spalled off from the interface and diminished, while the Ni_3_Sn_4_ layer (~2 µm) with a rod-like grain structure became more continuous and thicker. Moreover, after 5 min of reaction (Figure 2c), the (Pd,Ni)Sn_4_ completely disappeared, and the Ni_3_Sn_4_ phase became the primary interfacial product. Based on its microstructural characteristics, the Ni_3_Sn_4_ layer can be divided into two distinct regions. The region adjacent to the solder side consists of columnar Ni_3_Sn_4_ grains, whereas a continuous and dense layered structure is observed near the Ni substrate. The columnar Ni_3_Sn_4_ grains are mainly formed by precipitation during the early stage of dissolution of the Ni substrate into the molten solder, while the dense layered Ni_3_Sn_4_ region is formed through a diffusion-controlled reaction, in which Sn atoms diffuse toward the Ni substrate and react to form Ni_3_Sn_4_.

As shown in Figure 2d, after 30 min of reaction, the overall morphology of the Ni_3_Sn_4_ phase remained similar to that observed at shorter reaction times. However, the IMC layer became noticeably thicker, and the columnar grains exhibited pronounced coarsening. Notably, large faceted Ni_3_Sn_4_ grains were observed at the interface, which are likely transformed from the previously formed plate-like (Pd,Ni)Sn_4_ grains, as will be discussed in detail later.

#### 3.1.2. Sn (50 µm)/Au/Pd (200 nm)/Ni System

**Figure 3:**
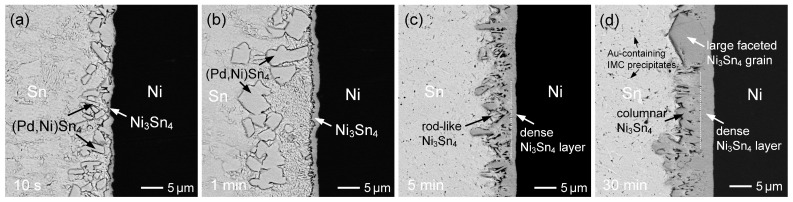
BSE micrographs showing the interfacial microstructures in the Sn (50 µm)/Au/Pd (200 nm)/Ni samples reacted at 260 °C for (**a**) 10 s, (**b**) 1 min, (**c**) 5 min, and (**d**) 30 min.

Figure 3 shows the interfacial microstructures of the Sn (50 µm)/Au/Pd (200 nm)/Ni system reacted at 260 °C for different reaction times. The evolution of interfacial phases is similar to that observed in Figure 2, including the initial formation of (Pd,Ni)Sn_4_ at short reaction times and the subsequent dominance of the Ni_3_Sn_4_ phase with prolonged reaction. However, notable differences in phase stability and microstructural development are observed due to the increased Pd thickness.

At the early stage (10 s and 1 min; Figure 3a,b), plate-like (Pd,Ni)Sn_4_ grains were more abundant at the interface than in the 70 nm Pd case, indicating that higher Pd content promoted their formation and temporary retention. Nevertheless, these large (Pd,Ni)Sn_4_ grains gradually spalled from the interface. As the reaction time increased to 5 min (Figure 3c), Ni_3_Sn_4_ became the dominant interfacial phase, forming a dual-layered structure with rod-like or columnar grains on the solder side and a dense layer adjacent to the Ni substrate, similar to that shown in Figure 2c. After 30 min of reaction (Figure 3d), the Ni_3_Sn_4_ layer became noticeably thicker, and the columnar grains exhibited significant coarsening. In addition, large faceted Ni_3_Sn_4_ grains were also observed, resembling those in Figure 2d.

Moreover, as shown in Figure 3d, fine and relatively bright filament-like precipitates were observed in the solder matrix. Although accurate compositional analysis of these fine IMCs was not feasible, contrast features and EPMA results indicate the presence of Au, suggesting that these precipitates are Au-containing IMCs, likely AuSn_4_ or (Au,Pd,Ni)Sn_4_. In the Sn/Au/Pd/Ni metallization system, the extremely thin Au layer rapidly dissolved into the molten solder during soldering. Upon cooling and solidification, the dissolved Au was transported away from the interface and participated in the formation of Au-containing IMC precipitates within the solder.

#### 3.1.3. Sn (50 µm)/Au/Pd (1 µm)/Ni System

**Figure 4:**
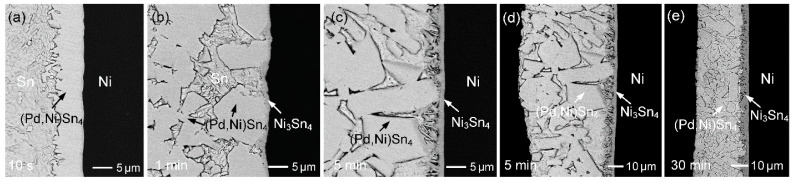
BSE micrographs showing the interfacial microstructures of Sn (50 µm)/Au/Pd (1 µm)/Ni samples reacted at 260 °C for (**a**) 10 s, (**b**) 1 min, (**c**) 5 min, (**d**) 5 min (low-magnification image), and (**e**) 30 min.

When the Pd thickness was increased to 1 µm, the interfacial microstructures at different reaction times were analyzed, and the results are shown in Figure 4a–e. After 10 s of reaction (Figure 4a), a thick and uniform IMC layer with a thickness of approximately 7 µm was formed at the interface. EPMA analysis identified this IMC as PdSn_4_, with no detectable Ni content, indicating that the Ni substrate had not yet been involved in the interfacial reaction at this stage. After 1 min of reaction (Figure 4b), a large amount of (Pd,Ni)Sn_4_ was formed, accompanied by the appearance of a thin Ni_3_Sn_4_ layer adjacent to the Ni substrate. With further reaction (Figure 4c), the (Pd,Ni)Sn_4_ phase gradually detached from the Ni_3_Sn_4_ layer, while the Ni_3_Sn_4_ phase exhibited a rod-like morphology. Surprisingly, nearly the entire Sn solder region was filled with (Pd,Ni)Sn_4_ grains (Figure 4d,e), demonstrating that the substantially increased Pd thickness strongly promoted the formation and persistence of the (Pd,Ni)Sn_4_ phase throughout the solder matrix. In addition, EPMA analysis revealed that the Ni content in the (Pd,Ni)Sn_4_ phase reached as high as 16.2 at.%.

**Figure 5:**
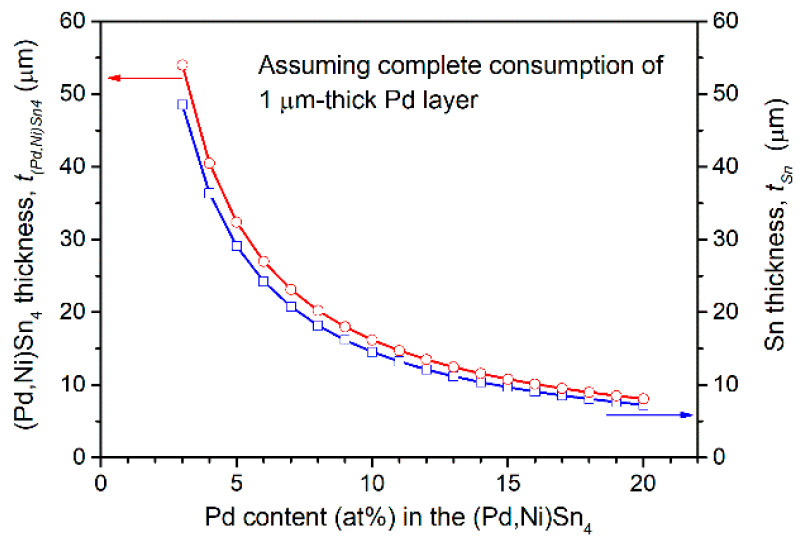
Estimated thickness of the formed (Pd,Ni)Sn_4_ (*t_(Pd,Ni)Sn_*_4_, red line) and the required Sn (*t_Sn_*, blue line) as a function of Pd atomic percentage, assuming complete consumption of a 1 µm thick Pd layer.

Although the Pd plating thickness was only 1 µm, it resulted in the extensive formation of (Pd,Ni)Sn_4_, which nearly filled the entire solder region. Such excessive IMC formation is known to induce embrittlement of solder joints, thus degrading their mechanical reliability. To quantitatively evaluate the extent of Pd consumption and its contribution to IMC formation, the theoretical thickness of PdSn_4_ produced from the Pd layer was estimated based on mass conservation and stoichiometric considerations. Assuming complete consumption of the 1 µm thick Pd layer, approximately 8.1 µm of PdSn_4_ can be formed, as detailed in the Supplementary Material. Moreover, Ni atoms can substitute for Pd sites in the PdSn_4_ lattice, resulting in a high Ni solubility of up to 16 at.% in the IMC. Therefore, the IMC thickness increases as Pd is partially replaced by Ni. When the Ni content reaches 10 at.%, the theoretical thickness of the resulting (Pd,Ni)Sn_4_ phase can increase to nearly twice that of stoichiometric PdSn_4_, reaching ~16 µm. Figure 5 illustrates the calculated thickness of (Pd,Ni)Sn_4_ as a function of Pd atomic percentage, assuming complete consumption of the 1 µm thick Pd layer. As the Pd content decreases to 4 at.% (i.e., forming (Pd_0.2_Ni_0.8_)Sn_4_), the thickness of the (Pd,Ni)Sn_4_ phase is theoretically predicted to increase to approximately 40 µm. This theoretical prediction shows good agreement with the experimental results (Figure 4e). Therefore, the extensive formation of (Pd,Ni)Sn_4_ can be reasonably explained by the high incorporation of Ni into the PdSn_4_ lattice, which significantly promotes the formation of the (Pd,Ni)Sn_4_ phase. In addition, Pd layers of 70 nm and 200 nm are estimated to react with approximately 2.6 and 7.4 µm of Sn, respectively, yielding about 2.8 and 8 µm of (Pd_0.2_Ni_0.8_)Sn_4_.

#### 3.1.4. Sn (20 µm)/Au/Pd (200 nm)/Ni System

**Figure 6:**
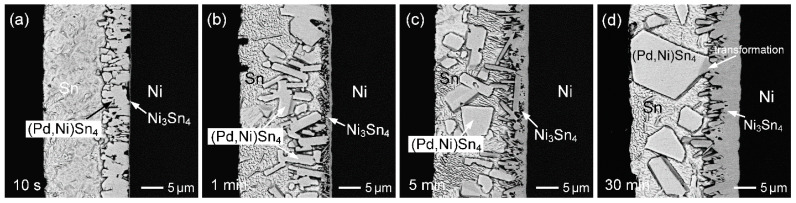
BSE micrographs showing the interfacial microstructures of Sn (20 µm)/Au/Pd (200 nm)/Ni samples reacted at 260 °C for (**a**) 10 s, (**b**) 1 min, (**c**) 5 min, and (**d**) 30 min.

The Sn layer thickness was reduced to 20 µm. Figure 6 presents BSE micrographs of the interfacial microstructures in the Sn (20 µm)/Au/Pd (200 nm)/Ni system reacted at 260 °C for various reaction times. During the initial reaction stage (10 s), a markedly thick (Pd,Ni)Sn_4_ layer was observed, with a Ni content of approximately 13 at.%, while an extremely thin Ni_3_Sn_4_ layer simultaneously formed at the (Pd,Ni)Sn_4_/Ni interface. At this stage, although Pd had dissolved and reacted to form (Pd,Ni)Sn_4_, the IMC layer remained attached to the interface and had not yet detached into the solder matrix. Following 1 min of reaction (Figure 6b), (Pd,Ni)Sn_4_ gradually delaminated from the interface. Simultaneously, Ni continued to diffuse from the substrate, modifying the local composition at the interface and driving the subsequent growth of Ni_3_Sn_4_ with a rod-like morphology.

As the reaction proceeded, the (Pd,Ni)Sn_4_ phase also underwent grain coarsening. As shown in Figure 6d, (Pd,Ni)Sn_4_ exhibited a large faceted grain morphology, with grain sizes exceeding 10 µm. A large faceted (Pd,Ni)Sn_4_ grain was observed to be attached to the Ni side of the interface, where partial transformation into Ni_3_Sn_4_ can be clearly identified. This observation suggests that with prolonged reaction time, continuous Ni diffusion from the substrate increases the local Ni concentration within the (Pd,Ni)Sn_4_ phase. Once the Ni content exceeds the solubility limit of (Pd,Ni)Sn_4_, the phase becomes thermodynamically unstable and may gradually transform into Ni_3_Sn_4_ as Ni continues to diffuse into its grains. Accordingly, this provides a reasonable explanation for the formation of the large faceted Ni_3_Sn_4_ grains observed in Figure 2d and Figure 3d, which originated from the faceted (Pd,Ni)Sn_4_ phase.

#### 3.1.5. Sn (20 µm)/Au/Pd (1 µm)/Ni System

**Figure 7:**
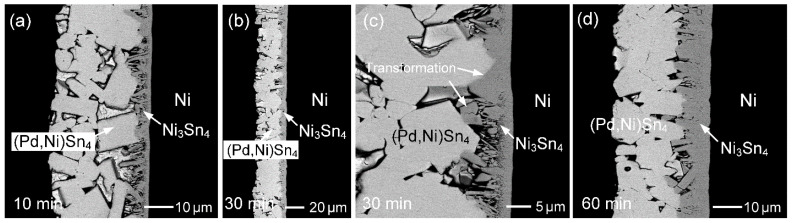
BSE micrographs showing the interfacial microstructures of Sn (20 µm)/Au/Pd (1 µm)/Ni samples reacted at 260 °C for (**a**) 10 min, (**b**) 30 min (low-magnification image), (**c**) 30 min, and (**d**) 60 min.

As illustrated in Figure 5 and Table S2, when a 1 µm thick Pd layer reacts with Sn and Ni to form (Pd,Ni)Sn_4_, decreasing the Pd content in the phase from 15 to 4 at.% results in an increase in the required Sn thickness from approximately 10 to 37 µm, while the corresponding thickness of the formed (Pd,Ni)Sn_4_ increases from about 11 to 40 µm. Accordingly, the Sn (20 µm)/Au/Pd (1 µm)/Ni reaction was carried out, as shown in Figure 7a–d. After 10 min of reaction, the interfacial microstructure was dominated by coarse, faceted (Pd,Ni)Sn_4_ grains adjacent to the Ni substrate, accompanied by a thin Ni_3_Sn_4_ layer on the Ni side. At this stage, the solder layer was largely consumed, with only about 10% of the Sn region remaining. With an increase in reaction time to 30 min (Figure 7b), the Sn layer was almost completely depleted, and the reaction zone was occupied primarily by large faceted (Pd,Ni)Sn_4_ grains. A higher-magnification image (Figure 7c) further reveals a partial transformation of (Pd,Ni)Sn_4_ to Ni_3_Sn_4_ near the Ni side. After 60 min of reaction (Figure 7d), the transformation became more pronounced, with no Sn remaining. The interfacial region was increasingly dominated by Ni_3_Sn_4_, while the amount of (Pd,Ni)Sn_4_ further decreased. This microstructural evolution indicated that (Pd,Ni)Sn_4_ served as a transitional phase, gradually converting into the thermodynamically more stable Ni_3_Sn_4_ under continuous Ni diffusion. These observations are consistent with the predictions shown in Figure 5.

#### 3.1.6. Sn (10 µm)/Au/Pd (70 nm)/Ni System

**Figure 8:**
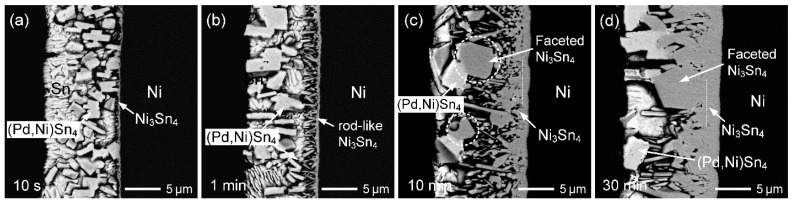
BSE micrographs showing the interfacial microstructures of Sn (10 µm)/Au/Pd (70 nm)/Ni samples reacted at 260 °C for (**a**) 10 s, (**b**) 1 min, (**c**) 10 min, and (**d**) 30 min.

The Sn layer thickness was further reduced to 10 µm. Figure 8a–d show the reactions of Sn (10 µm)/Au/Pd (70 nm)/Ni at different times. After 10 s of reaction, a large number of (Pd,Ni)Sn_4_ grains were dispersed throughout the solder matrix. After 1 min, the (Pd,Ni)Sn_4_ grains coarsened, and rod-like Ni_3_Sn_4_ phases became clearly visible. With further reaction up to 10 min, the interfacial region was predominantly composed of Ni_3_Sn_4_, and several large faceted Ni_3_Sn_4_ grains were observed (highlighted by white dashed circles). As discussed previously, these faceted grains originated from the transformation of (Pd,Ni)Sn_4_ grains. When extending to 30 min, the IMC almost converted to Ni_3_Sn_4_.

**Figure 9:**
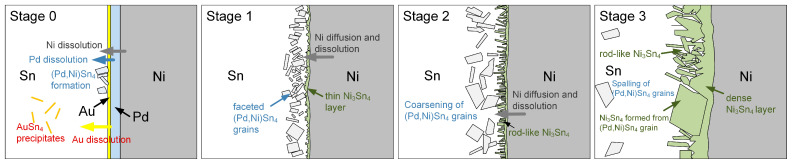
Schematic illustration of the microstructural evolution and reaction mechanism during the Sn/Au/Pd/Ni interfacial reactions.

The formation of reaction phases and the associated microstructural evolution are strongly influenced by the thicknesses of the Pd and Sn layers, as well as their thickness ratio. Based on the above observations, the interfacial reaction can be categorized into three distinct stages, as illustrated in Figure 9.

Stage I: Initial Au and Pd dissolution and (Pd,Ni)Sn_4_ formation

At the early reaction stage, the thin Au and Pd layers rapidly dissolved into molten Sn, resulting in the formation of fine AuSn_4_ and (Pd,Ni)Sn_4_ grains dispersed in the solder matrix near the interface. Additionally, a very thin Ni_3_Sn_4_ layer was formed at the interface.

Stage II: Concurrent formation of (Pd,Ni)Sn_4_ and Ni_3_Sn_4_ grains

With increasing reaction time, continued Ni diffusion enriched the local Ni concentration of the solder near the interface, resulting in the nucleation of rod-like Ni_3_Sn_4_ and the coarsening of (Pd,Ni)Sn_4_ grains. This observation indicates the simultaneous formation and parallel evolution of the two IMCs at the solder/Ni interface.

Stage III: Transformation of (Pd,Ni)Sn_4_ into thermodynamically stable Ni_3_Sn_4_

At prolonged reaction times, the interfacial region became predominantly composed of Ni_3_Sn_4_, as the pre-existing faceted (Pd,Ni)Sn_4_ grains were progressively replaced by Ni_3_Sn_4_ under a continuous supply of Ni. This evolution reflects the higher thermodynamic stability of Ni_3_Sn_4_ under Ni-rich interfacial conditions.

Moreover, as the solder thickness decreases, the reaction progression is markedly accelerated. Consequently, with the miniaturization of solder joints, interfacial reactions and microstructural evolution proceed at an increasingly rapid rate.

At the solder/Ni interface, distinct diffusion fluxes of Pd, Au, and Ni can be inferred from the interfacial microstructural evolution, as illustrated in Figure 9. During the early stage of soldering, Au from the extremely thin Au layer (~20 nm) rapidly dissolves into the molten solder, resulting in a dominant Au flux away from the interface. Upon cooling, this dissolved Au is transported into the solder and precipitates as Au-containing IMCs. In contrast, Pd does not diffuse far into the solder but remains localized near the substrate interface. This behavior is attributed to its strong affinity for Ni, which promotes the formation of the thermodynamically stable (Pd,Ni)Sn_4_ phase. As a result, the Pd diffusion flux is effectively confined to the interfacial region and continuously consumed by (Pd,Ni)Sn_4_ formation.

The Ni flux originates from the Ni substrate and diffuses toward the solder through the interfacial IMC layers. At short reaction times, direct contact between Ni_3_Sn_4_ and the solder facilitates Ni dissolution, providing a sufficient Ni flux to support (Pd,Ni)Sn_4_ formation. Notably, with a thicker Pd layer (Figure 4 and Figure 7), the formation of a denser (Pd,Ni)Sn_4_ layer restricts Ni transport, leading to a reduced Ni flux toward the solder and consequently suppressing the growth of both (Pd,Ni)Sn_4_ and Ni_3_Sn_4_.

### 3.2. Sn-Pd/Ni Reactions

During the solder/Au/Pd/Ni reactions, the Pd layer dissolved into the solder, thereby increasing the Pd concentration in the molten Sn. For example, in the Sn (50 µm)/Pd/Ni system, complete dissolution of the Pd layer corresponds to estimated Pd contents of approximately 0.23 wt.% and 0.65 wt.% for Pd thicknesses of 70 nm and 200 nm, respectively. Accordingly, the Sn-Pd/Ni interfacial reactions were further investigated by varying the Pd content from 0.1 wt.% to 1 wt.%.

**Figure 10:**
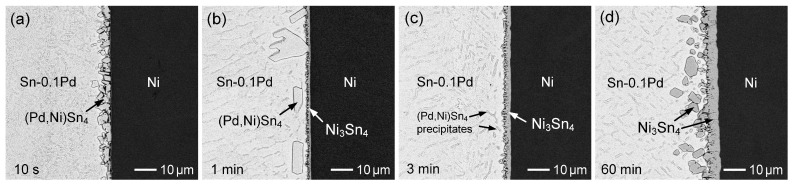
BSE micrographs showing the interfacial microstructures of Sn-0.1 wt.%Pd/Ni reaction at 260 °C for (**a**) 10 s, (**b**) 1 min, (**c**) 3 min, and (**d**) 60 min.

Figure 10a–d show the interfacial microstructures of the Sn-0.1 wt.%Pd/Ni reaction at 260 °C for reaction times ranging from 10 s to 60 min. After only 10 s of reaction (Figure 10a), numerous bright IMC grains formed continuously on the Ni substrate. EPMA analysis indicated a composition of Sn-4.3 at.%Pd-15.2 at.%Ni, identifying this phase as (Pd,Ni)Sn_4_ with a high Ni solubility. After 1 min of reaction (Figure 10b), the (Pd,Ni)Sn_4_ phase exhibited a large faceted grain morphology; however, its overall amount decreased, and some spalled into the solder. In contrast, the Ni_3_Sn_4_ phase was observed on the Ni substrate. When the reaction increased to 3 min (Figure 10c), the (Pd,Ni)Sn_4_ phase completely disappeared, leaving only a continuous Ni_3_Sn_4_ layer at the interface. With further reaction, the Ni_3_Sn_4_ phase remained stable and continued to thicken on the Ni substrate. Upon prolonging the reaction to 60 min (Figure 10d), the Ni_3_Sn_4_ grains became unstable and gradually spalled into the molten Sn.

Based on the above microstructural evolution and the assumption of local equilibrium in the interfacial zone, it can be inferred that, at the initial stage of reaction, a small amount of Ni dissolved from the substrate into the solder, thus increasing the local Ni concentration near the interface. This promoted the rapid precipitation of Pd and Ni from the solder, leading to the formation of the (Pd,Ni)Sn_4_ phase. With continued Ni dissolution into the solder, the interfacial Ni concentration further increased, driving a phase transition toward Ni_3_Sn_4_ as the thermodynamically stable interfacial phase. Meanwhile, although the (Pd,Ni)Sn_4_ grains coarsened, they gradually re-dissolved into the solder and eventually disappeared.

**Figure 11:**
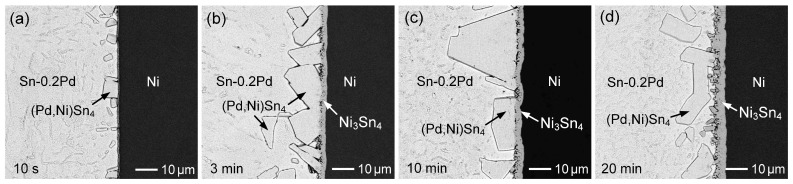
BSE micrographs showing the interfacial microstructures of Sn-0.2 wt.%Pd/Ni reaction at 260 °C for (**a**) 10 s, (**b**) 3 min, (**c**) 10 min, and (**d**) 20 min.

Furthermore, when the Pd content was increased to 0.2 wt.%, the corresponding microstructural evolution is shown in Figure 11a–d. Similar to the Sn-0.1 wt.% Pd case, (Pd,Ni)Sn_4_ grains formed on the Ni substrate after 10 s of reaction. When the reaction time increased to 1 and 3 min, the (Pd,Ni)Sn_4_ phase remained stably attached to the underlying Ni_3_Sn_4_ layer. Based on the microstructural observations, the formation of (Pd,Ni)Sn_4_ could be attributed to the diffusion of Pd atoms in the solder toward the Ni substrate, which is driven by the attractive interaction with Ni. This Ni-induced Pd diffusion facilitates interfacial reactions and promotes the formation of the (Pd,Ni)Sn_4_ phase, rather than simple precipitation caused by Ni dissolution into the solder, leading to supersaturation of Ni and Pd in the solder. After 20 min (Figure 11d), the faceted (Pd,Ni)Sn_4_ grains gradually spalled into the molten solder. After a prolonged reaction time of 60 min, large faceted (Pd,Ni)Sn_4_ grains were observed to remain present while being dispersed near the interface. This observation indicated that increasing the Pd content from 0.1 to 0.2 wt.% enhanced the stability of the (Pd,Ni)Sn_4_ phase, enabling its coexistence with Ni_3_Sn_4_.

**Figure 12:**
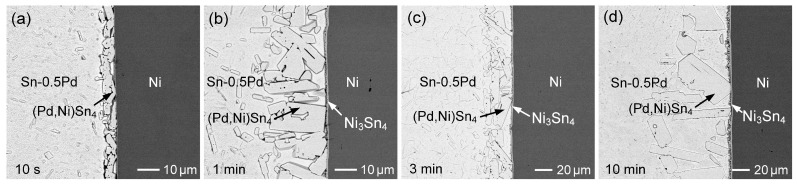
BSE micrographs showing the interfacial microstructures of Sn-0.5 wt.%Pd/Ni reaction at 260 °C for (**a**) 10 s, (**b**) 1 min, (**c**) 3 min, and (**d**) 10 min.

**Figure 13:**
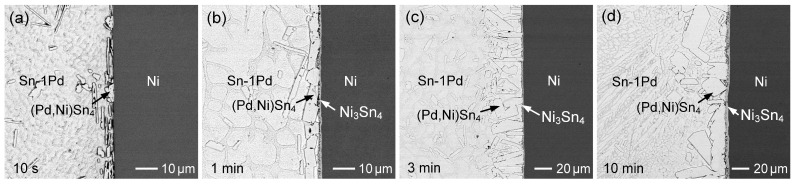
BSE micrographs showing the interfacial microstructures of Sn-1 wt.%Pd/Ni reaction at 260 °C for (**a**) 10 s, (**b**) 1 min, (**c**) 3 min, and (**d**) 10 min.

When the Pd concentration was increased to 0.5 wt.% and 1 wt.%, the interfacial results are shown in Figure 12 and Figure 13. A substantial amount of (Pd,Ni)Sn_4_ formed at the interface, likely due to Pd diffusion toward the Ni-rich region. After 3 min, these (Pd,Ni)Sn_4_ grains aggregated into a relatively dense and continuous IMC phase. Compositional analysis revealed higher Ni content (~16 at.%) in the (Pd,Ni)Sn_4_ adjacent to the Ni substrate, compared to ~8–12 at.% on the opposite side. A Ni_3_Sn_4_ layer was also observed but remained thin. Its growth was limited, likely due to the adherence of (Pd,Ni)Sn_4_, which favored Ni diffusion into the (Pd,Ni)Sn_4_ phase until its solubility limit was reached, thereby restricting the Ni_3_Sn_4_ growth.

**Figure 14:**
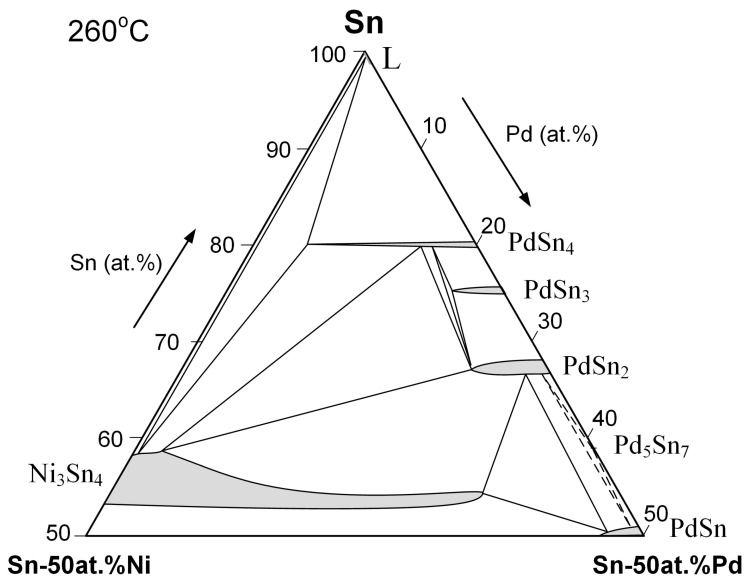
Proposed partial isothermal phase diagram of the Sn-Pd-Ni system at 260 °C in the Sn-rich region, constructed from literature data [25,27] and the interfacial (Pd,Ni)Sn_4_ compositions obtained in this study. The gray areas represent single-phase regions and the dotted lines represent estimated phase regions.

Jandl and Richter [27] investigated the Sn-rich corner of the Sn-Pd-Ni ternary system and reported an isothermal phase diagram at 250 °C. According to their experimental results, the maximum equilibrium solubility of Ni in the (Pd,Ni)Sn_4_ phase was 11.6 at.%, which is lower than the 15.4 at.% Ni reported by Rahman et al. [25]. In the present study, the interfacial (Pd,Ni)Sn_4_ phase formed under non-equilibrium interfacial reaction conditions was found to contain up to approximately 16 at.% Ni. Consequently, based on the combined results of the above-mentioned studies, a partial isothermal phase diagram of the Sn-Pd-Ni system is proposed, as illustrated in Figure 14.

Based on the hypothesis of local interfacial phase equilibrium, the reaction phases formed at the interface correspond to the equilibrium phases in the phase diagram. In the Sn-0.1Pd/Ni system, the (Pd,Ni)Sn_4_ phase initially formed, but rapidly transformed into Ni_3_Sn_4_ as the dominant phase due to increasing Ni concentration in the solder. The interfacial reaction phases and microstructural evolution closely reflect the transitions between equilibrium phase regions. Similarly, in the Sn-0.2Pd/Ni system, (Pd,Ni)Sn_4_ appeared at the early reaction stage, indicating phase equilibrium between Sn-0.2Pd and (Pd,Ni)Sn_4_. After 3 min, (Pd,Ni)Sn_4_ coexisted with Ni_3_Sn_4_ at the interface, corresponding to the L-(Pd,Ni)Sn_4_-Ni_3_Sn_4_ tie triangle in the phase diagram. With a prolonged reaction time (60 min), (Pd,Ni)Sn_4_ grains dispersed from the interface, leaving Ni_3_Sn_4_ as the sole interfacial phase, reflecting a further shift in the equilibrium toward Ni_3_Sn_4_. The Sn-0.5Pd/Ni and Sn-1Pd/Ni systems showed a similar trend, but higher Pd content delayed the phase transformation, extending the timescale of the evolution.

**Figure 15:**
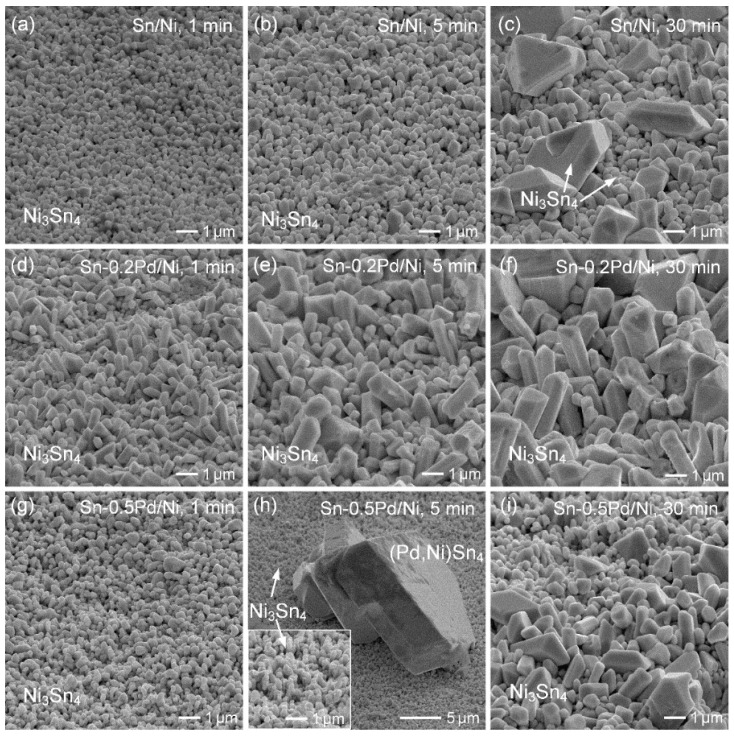
Top-view SEM images showing the grain morphologies of Ni_3_Sn_4_ formed on Ni substrates after reaction with (**a**–**c**) pure Sn, (**d**–**f**) Sn–0.2Pd, and (**g**–**i**) Sn–0.5Pd solders for 1, 5, and 30 min.

Moreover, Figure 15 shows the grain morphologies of Ni_3_Sn_4_ formed on Ni substrates after reacting with pure Sn, Sn-0.2Pd, and Sn-0.5Pd solders for 1, 5, and 30 min. For the Sn/Ni system, Ni_3_Sn_4_ exhibited a fine-grained morphology at the initial reaction stage, which progressively coarsened into large faceted crystals with prolonged reaction time, reflecting rapid Ni diffusion and continuous Ni_3_Sn_4_ growth. After 30 min of reaction, another type of large faceted, chunk-like Ni_3_Sn_4_ grains was observed. A similar microstructure was reported in a previous study [28], which was attributed to the locally high Ni concentration near the interface, with the formation of large faceted grains governed by the dissolution and subsequent reprecipitation of Ni_3_Sn_4_.

With the addition of 0.2 wt.% Pd, the Ni_3_Sn_4_ remained dominated. Compared to the Pd-free sample, the Ni_3_Sn_4_ grains exhibited a more columnar morphology. This columnar structure is primarily attributed to Pd-enhanced nucleation at the interface, which restricted lateral growth and promoted preferential growth perpendicular to the interface. In the Sn-0.5 wt.% Pd/Ni system, fine Ni_3_Sn_4_ grains were observed at the early stage (1 min), indicating that Pd further enhanced Ni_3_Sn_4_ nucleation. Although faceted (Pd,Ni)Sn_4_ grains also formed initially, most were removed during deep etching and ultrasonic agitation, leaving primarily Ni_3_Sn_4_ for observation. In the 5 min sample, when the solder was not completely removed, large faceted (Pd,Ni)Sn_4_ grains were clearly observed together with Ni_3_Sn_4_, demonstrating that Pd strongly participated in the interfacial reaction and temporarily stabilized the (Pd,Ni)Sn_4_ phase. With further reaction (30 min), the interfacial microstructure again became dominated by Ni_3_Sn_4_, with columnar grains accompanied by noticeable grain coarsening. These results reasonably account for the rod-like morphology of Ni_3_Sn_4_ observed in the Sn/Au/Pd/Ni samples, as shown in Figure 2b,c, which can be attributed to Pd-enhanced nucleation and preferential growth normal to the interface.

### 3.3. Sn-3Ag-Pd/Ni Reactions

**Figure 16:**
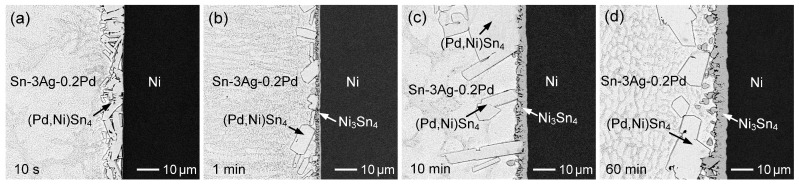
BSE micrographs showing the interfacial microstructures of Sn-3Ag-0.2Pd/Ni reaction at 260 °C for (**a**) 10 s, (**b**) 1 min, (**c**) 10 min, and (**d**) 60 min.

Similar interfacial reactions between Ni and Sn-3Ag-Pd solders with various Pd contents (0.1–1 wt.%) were investigated. Figure 16a–d show the interfacial microstructures of the Sn-3Ag-0.2Pd/Ni couple reacted at 260 °C for 10 s, 1 min, 10 min, and 60 min, respectively. The (Pd,Ni)Sn_4_ phase initially formed at the interface and subsequently coexisted with Ni_3_Sn_4_ after 1 min and 10 min of reaction. With prolonged reaction time, the (Pd,Ni)Sn_4_ grains gradually coarsened, accompanied by continuous thickening of the Ni_3_Sn_4_ layer. After 60 min, large faceted (Pd,Ni)Sn_4_ grains spalled from the interface. These results are similar to those observed in the Sn-0.2Pd/Ni system, indicating that the presence of Ag in the solder does not significantly affect the interfacial reaction behavior.

**Figure 17:**
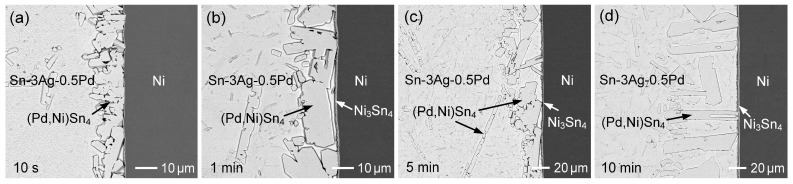
BSE micrographs showing the interfacial microstructures of Sn-3Ag-0.5Pd/Ni reaction at 260 °C for (**a**) 10 s, (**b**) 1 min, (**c**) 5 min, and (**d**) 10 min.

The Sn-3Ag-0.1Pd/Ni reaction was also examined, as shown in Figure S1. Owing to the reduced Pd content (0.1 wt.%), Ni_3_Sn_4_ became the dominant interfacial reaction phase. When the Pd content was increased to 0.5 wt.%, the corresponding interfacial reaction results are presented in Figure 17a–d. At the early reaction stage, an irregular (Pd,Ni)Sn_4_ layer with a thickness of ~15 µm formed at the interface, consisting of numerous faceted grains. A similar microstructure was also observed in the Sn-3Ag-0.2Pd/Ni system (Figure 16a). As discussed previously, during the initial reaction stage, the Ni substrate dissolved into the molten solder, resulting in a relatively high Ni concentration in the vicinity of the interface. The strong interaction between Ni and Pd promoted the formation of the thermodynamically stable (Pd,Ni)Sn_4_ phase, leading to local depletion of Pd at the interface and driving Pd diffusion from the solder toward the interface. Consequently, a large amount of (Pd,Ni)Sn_4_ formed rapidly at the interface within a very short reaction time. With increasing reaction time, continuous Ni diffusion from the substrate enriches the interfacial region with Ni, leading to the concurrent formation and growth of the Ni_3_Sn_4_ phase.

**Figure 18:**
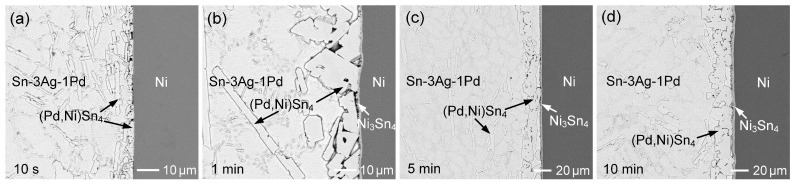
BSE micrographs showing the interfacial microstructures of Sn-3Ag-1Pd/Ni reaction at 260 °C for (**a**) 10 s, (**b**) 1 min, (**c**) 5 min, and (**d**) 10 min.

Furthermore, the interfacial reaction of the Sn-3Ag-1Pd solder was investigated, as shown in Figure 18. Notably, at the initial reaction stage (10 s), the interfacial phase exhibited a stripe-like microstructure with a relatively low Ni content in (Pd,Ni)Sn_4_, below 5 at.%. Pure PdSn_4_ typically forms a plate-like three-dimensional morphology, which appears as a stripe-like feature in two-dimensional metallographic observations. A similar stripe-like morphology was also observed in the Sn-1 Pd/Ni sample at 10 s (Figure 13a). When the Pd concentration was increased to 1 wt.%, the initially formed IMC phase still corresponded to low-Ni (Pd,Ni)Sn_4_. With reaction time extended to 1 min (Figure 13b and Figure 18b), significant Ni dissolution from the substrate occurred at the interface, resulting in the transformation of the IMC phase into faceted grains with a higher Ni content in (Pd,Ni)Sn_4_. In addition, compared with lower-Pd systems, the higher Pd content in the solder stabilized the (Pd,Ni)Sn_4_ phase and retarded its transformation, resulting in the prolonged coexistence of (Pd,Ni)Sn_4_ and Ni_3_Sn_4_ at the interface. These results indicate that Pd not only influenced the early-stage microstructure but also modulated the interfacial phase evolution.

**Figure 19:**
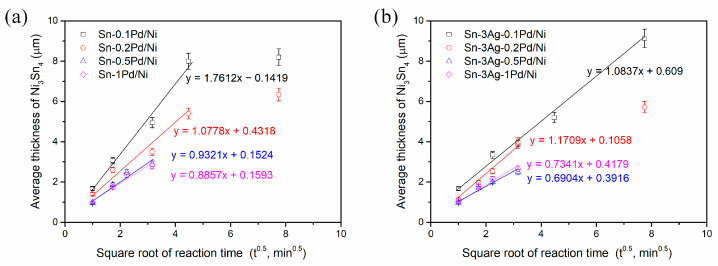
Average thickness of the Ni_3_Sn_4_ layer as a function of the square root of reaction time for (**a**) Sn-Pd/Ni and (**b**) Sn-3Ag-Pd/Ni systems at 260 °C with different Pd contents.

From the reaction results of the Sn-Pd/Ni and Sn-3Ag-Pd/Ni systems, it can be observed that the thickness of the formed Ni_3_Sn_4_ layer was influenced by the Pd content. Figure 19a,b present the average thickness of the Ni_3_Sn_4_ IMC layer formed at the solder/Ni interface in the Sn-Pd and Sn-3Ag-Pd systems, respectively. In both systems, the IMC thickness (*x*) increased linearly with the square root of reaction time (*t*^0.5^), indicating that the growth of the Ni_3_Sn_4_ layer was primarily governed by diffusion-controlled kinetics. This behavior can be described by the parabolic growth law:

*x* = *k*·*t*^0.5^ (1)

where *x* is the IMC thickness, *t* is the reaction time, and *k* is the growth rate constant. The slope of the linear fitting of *x* versus *t*^0.5^ corresponds directly to *k*, providing a quantitative measure of the reaction kinetics. This behavior was consistent with the previous study on the Sn/Ni interfacial reactions [29], which reported that during the very early stage (e.g., within the first 2.5 min at 250 °C), Ni_3_Sn_4_ grains grew proportionally to *t*^1/3^ before transitioning to a parabolic relationship at longer reaction times.

For the Sn-Pd/Ni system (Figure 19a), the Ni_3_Sn_4_ layer formed in the Sn-0.1Pd/Ni system was relatively thicker and exhibited a steeper fitting slope, indicating a higher growth rate. With increasing Pd content to 0.2 wt.% and 0.5 wt.%, the thicknesses of the Ni_3_Sn_4_ layers decreased, and the corresponding growth rates became slower, while comparable thicknesses and growth rates were observed for the Sn-0.5Pd/Ni and Sn-1Pd/Ni systems. As shown in Figure 10, Figure 11, Figure 12 and Figure 13, when the Pd content exceeded 0.2 wt.%, the (Pd,Ni)Sn_4_ grains were formed above the Ni_3_Sn_4_ layer, suggesting that increased Pd promoted (Pd,Ni)Sn_4_ formation and consumed part of the Ni flux from the Ni substrate. As a result, the effective Ni supply available for Ni_3_Sn_4_ formation was reduced, thus suppressing Ni_3_Sn_4_ growth.

A similar diffusion-controlled growth trend was observed in the Sn-3Ag-Pd/Ni system (Figure 19b). Compared with the Ag-free solder, the growth rates were relatively lower, indicating that Ag addition also reduced the Ni_3_Sn_4_ formation. Furthermore, similar to the Sn-Pd/Ni system, increasing the Pd content further suppressed Ni_3_Sn_4_ growth. As shown in Figure 16, Figure 17 and Figure 18, when the Pd concentration exceeded 0.2 wt.%, the (Pd,Ni)Sn_4_ was also formed. In particular, for 0.5 wt.% and 1 wt.% Pd, a larger amount of (Pd,Ni)Sn_4_ developed a dense layered structure attached to Ni_3_Sn_4_, significantly hindering its growth.

It was noted that the amount of (Pd,Ni)Sn_4_ formed at the solder/Ni interface initially increased when the Pd content in the solder rose from 0.2 wt.% to 0.5 wt.% (Figure 11, Figure 12, Figure 13, Figure 16, Figure 17 and Figure 18). At this intermediate Pd level, the higher Pd content promoted (Pd,Ni)Sn_4_ formation, and the grains were generally large and loosely attached to the interface. This allowed Ni_3_Sn_4_ to remain in contact with the solder, enabling partial dissolution or diffusion of Ni into the molten solder, which provided a Ni flux that further stabilized and enhanced the (Pd,Ni)Sn_4_ growth.

In contrast, for the 1 wt.% Pd case, the (Pd,Ni)Sn_4_ grains formed after 10 s and 1 min were smaller and more densely packed. Compositional analysis indicated a lower Ni content (<10 at.%) in these grains. The dense (Pd,Ni)Sn_4_ grains adhered closely to the Ni substrate, forming the (Pd,Ni)Sn_4_/Ni_3_Sn_4_/Ni interfacial structure. This microstructure restricted Ni supply from the substrate, reducing the overall (Pd,Ni)Sn_4_ formation. Furthermore, the dense grains limited direct contact between Ni_3_Sn_4_ and the solder, suppressing the Ni_3_Sn_4_ growth. These observations indicated that the formation and growth of (Pd,Ni)Sn_4_ strongly depend on both the Pd concentration in the solder and the availability of Ni flux at the interface.

## 4. Conclusions

The effects of Pd thickness and Pd concentration in solder on interfacial reactions with Ni were systematically investigated at 260 °C. Au/Pd/Ni substrates with Pd layers of 70 nm, 200 nm, and 1 µm were reacted with electroplated Sn layers of varying thicknesses to evaluate the influence of Pd supply from surface finishes. In parallel, Sn-Pd and Sn-3Ag-Pd solders containing 0.1–1 wt.% Pd were reacted with Ni to examine the effect of Pd concentration in molten solder. The results demonstrate that Pd availability strongly governs interfacial phase formation and microstructural evolution. With thin Pd layers or low Pd contents, the initially formed (Pd,Ni)Sn_4_ phase was transient and rapidly replaced by Ni_3_Sn_4_ as Ni diffusion from the substrate. Theoretical calculations indicate that Pd consumption, accompanied by Ni supply, leads to a pronounced (Pd,Ni)Sn_4_ formation. Specifically, 70 nm, 200 nm, and 1 µm Pd layers are predicted to consume approximately 2.6, 7.4, and 36.7 µm of Sn, respectively, resulting in the formation of about 2.8, 8, and 40 µm of (Pd_0.2_Ni_0.8_)Sn_4_. Therefore, increasing the Pd coating thickness or the Pd content in the solder markedly enhanced the formation, retention, and coarsening of (Pd,Ni)Sn_4_, resulting in its prolonged coexistence with Ni_3_Sn_4_. The transformation of (Pd,Ni)Sn_4_ into Ni_3_Sn_4_ occurred once the local Ni concentration exceeded its solubility limit. Pd also significantly influenced the grain morphology of Ni_3_Sn_4_. Minor Pd additions enhanced Ni_3_Sn_4_ nucleation, restricting lateral growth and promoting preferential growth normal to the interface, which resulted in refined, columnar (rod-like) Ni_3_Sn_4_ grains. Moreover, reducing Sn thickness accelerated interfacial reactions and phase evolution, highlighting the sensitivity of Pd-related interfacial phenomena to solder volume in miniaturized joints.

## Figures and Tables

**Table 1:** Summary of diffusion couples investigated in this study, including the Sn/Au/Pd/Ni, Sn-Pd/Ni, and Sn-3Ag-Pd/Ni systems, with corresponding solder compositions, solder thicknesses, and Pd layer thicknesses.

Sn/Au/Pd/Ni Systems	Sn-Pd/Ni Systems	Sn-3Ag-Pd/Ni Systems
Sn (50 µm)/Au/Pd (70 nm)/Ni	Sn-0.1 wt.%Pd	Sn-3Ag-0.1Pd/Ni (wt.%)
Sn (50 µm)/Au/Pd (200 nm)/Ni	Sn-0.2 wt.%Pd	Sn-3Ag-0.2Pd/Ni
Sn (50 µm)/Au/Pd (1 µm)/Ni	Sn-0.5 wt.%Pd	Sn-3Ag-0.5Pd/Ni
Sn (20 µm)/Au/Pd (200 nm)/Ni	Sn-1 wt.%Pd	Sn-3Ag-1Pd/Ni
Sn (20 µm)/Au/Pd (1 µm)/Ni		
Sn (10 µm)/Au/Pd (70 nm)/Ni		

## Data Availability

The original contributions presented in this study are included in the article/Supplementary Material. Further inquiries can be directed to the corresponding author.

## Supplementary Materials

The following supporting information can be downloaded at: https://www.mdpi.com/article/10.3390/ma19030526/s1, Figure S1. BSE micrographs showing the interfacial microstructures of Sn-3Ag-0.1Pd/Ni reaction at 260 °C for (a) 1 min, (b) 5 min, (c) 10 min, and (d) 60 min; Table S1. Atomic weight, density, and crystallographic data of Sn, Pd, and PdSn_4_; Table S2. Calculated thickness of the formed (Pd,Ni)Sn_4_ (*t_(Pd,Ni)Sn_*_4_) and the require Sn (*t_Sn_*) a function of Pd atomic percentage, assuming complete consumption of a 1 μm-thick Pd layer.
