# Supplementary material for: Influence of Pd Coating Thickness and Pd Content in Sn-Based Solders on Interfacial IMC Formation and Microstructural Evolution in Solder/Ni Joints
Source: Materials (Basel). 2026 Jan 28;19(3):526. doi: 10.3390/ma19030526 (PMC12898616; doi:10.3390/ma19030526)
Supplement: Supplementary file 1 [file materials-19-00526-s001.zip › materials-4117072-supplementary.pdf]

materials

Supplementary material

Influence of Pd Coating Thickness and Pd Content in Sn-Based Solders on Interfacial IMC Formation and Microstructural Evolution in Solder/Ni Joints

Chao-hong Wang*, Chu-an Li, Kuan-ting Li, and Hsuan-wei Chiu

Department of Chemical Engineering, National Chung Cheng University, Chiayi 621301, Taiwan
* Correspondence: chmchw@ccu.edu.tw

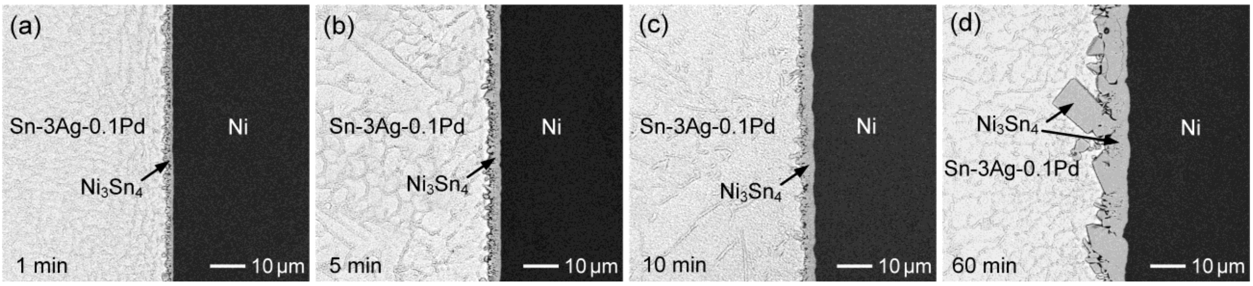

Figure S1. BSE micrographs showing the interfacial microstructures of Sn-3Ag-0.1Pd/Ni reaction at 260 °C for (a) 1 min, (b) 5 min, (c) 10 min, and (d) 60 min.

Estimation of the thickness of $PdSn_4$ formed from a Pd layer

Part I

The theoretical thickness of $PdSn_4$ formed from a Pd layer was estimated based on mass conservation and stoichiometric considerations. Assuming complete consumption of Pd and a uniform reaction over a unit area (A cm²), a Pd layer with a thickness of 1 μm (1 × 10⁻⁴ cm) has a volume of A × 10⁻⁴ cm³. According to the mass balance of Pd,

$$\mathrm{m}_{Pd} = 1\times 10^{-4}\times A\times \rho_{Pd} = t_{PdSn_4}\times 10^{-4}\times A\times \rho_{PdSn_4}\times \frac{MW_{Pd}}{MW_{PdSn_4}}$$

where MW_{PdSn4} and MW_{Pd} are the molecular weights of $PdSn_4$ (581.2 g mol^{-1}) and Pd (106.4 g mol^{-1}). Using the density of $PdSn_4$ and Pd (ρ_{PdSn4} = 8.17 g cm^{-3}, ρ_{Pd} = 12.008 g cm^{-3}), as seen in Table S1, the thickness of the resulting $PdSn_4$ layer (t_{PdSn4}) can be calculated as

$$t_{PdSn_4} = \frac{\rho_{Pd}}{\rho_{PdSn_4}}\times \frac{MW_{PdSn_4}}{MW_{Pd}} = \frac{12.008}{8.17}\times \frac{581.16}{106.4} = 8.03\ (\mu m)$$

This estimation indicates that a 1 μm-thick Pd layer can theoretically generate an ~8 μm-thick $PdSn_4$ layer if fully consumed during the interfacial reaction.

Part II

Ni atoms can substitute for Pd in the $PdSn_4$ lattice, forming $(Pd,Ni)Sn_4$.

Its composition can be expressed as $(Pd_X,Ni_{1-X})Sn_4$ in terms of atomic fraction or as $Pd_YNi_{20-Y}Sn_{80}$ in atomic percent (at.%).

According to the mass balance for Pd in the IMC, derive the relationship between $t_{(Pd,Ni)Sn4}$ and t_{PdSn4}.

$$t_{PdSn_4} = t_{(Pd,Ni)Sn_4}\times X = t_{(Pd,Ni)Sn_4}\times \frac{Y}{20}$$

$$t_{(Pd,Ni)Sn_4} = t_{PdSn_4}\cdot \frac{1}{X} = t_{PdSn_4}\cdot \frac{20}{Y}$$

The calculated results are summarized in Table S1. As the Pd content in the $(Pd,Ni)Sn_4$ phase decreases, indicating that more Pd lattice sites are replaced by Ni, the resulting $(Pd,Ni)Sn_4$ layer becomes thicker. Meanwhile, a larger amount of Sn is required (or consumed) to form the IMC. Here, t_{Sn} denotes the required thickness of the Sn layer. Using a 1-μm-thick Pd layer as the reference, the formation of $Pd_YNi_{20-Y}Sn_{80}$ was evaluated based on atomic ratio considerations. The required Sn thickness, t_{Sn}, is determined from the atomic ratio of Pd to Sn (Y : 80) as:

$$\frac{\rho_{Pd}\times 1\times 10^{-4}\times A}{MW_{Pd}}=\frac{\rho_{Sn}\times t_{Sn}\times 10^{-4}\times A}{MW_{Sn}}=Y:80$$

$$t_{Sn}=\frac{80}{Y}\frac{\rho_{Pd}}{\rho_{Sn}}\frac{MW_{Sn}}{MW_{Pd}}=\frac{80}{Y}\times\frac{12.008}{7.287}\times\frac{118.7}{106.4}=\frac{147.07}{Y}$$

The required Sn thickness was calculated for different Pd contents and is summarized in Table S2. The results are also plotted in Figure 5.

Table S1 Atomic weight, density, and crystallographic data of Sn, Pd, and $PdSn_4$.

Phase / JCPDS No.	Atomic weight	Density (g/cm³)	Crystal structure
Sn (89-4898)	118.69	7.287	BCT (body centered tetragonal)<br>S.G. $I4_1$/amd (141)<br>a = 5.831 Å<br>c = 3.182 Å
Pd (89-4897)	106.4	12.008	FCC (face-centered cubic)<br>S.G.: Fm$\bar{3}$m (225)<br>a = 6.397 Å
$PdSn_4$ (65-1402)	581.16	8.17	Orthorhombic<br>S.G.: Aba2 (41)<br>a = 6.397 Å<br>b = 6.425 Å<br>c = 11.495 Å

Table S2 Calculated thickness of the formed $(Pd,Ni)Sn_4$ ($t_{(Pd,Ni)Sn4}$)and the require Sn (t_{Sn}) a function of Pd atomic percentage, assuming complete consumption of a 1 μm-thick Pd layer.

Pd at.% (Y)	3	4	5	6	7	8	9	10	11
$t_{(Pd,Ni)Sn4}$ (μm)	53.53	40.15	32.12	26.77	22.94	20.07	17.84	16.06	14.6
t_{Sn} (μm)	49.02	36.77	29.41	24.51	21.01	18.38	16.34	14.71	13.37
Pd at.% (Y)	12	13	14	15	16	17	18	19	20
$t_{(Pd,Ni)Sn4}$ (μm)	13.38	12.35	11.47	10.71	10.04	9.45	8.92	8.45	8.03
t_{Sn} (μm)	12.26	11.31	10.51	9.80	9.19	8.65	8.17	7.74	7.35
